# Screening, Diagnosis and Management of Sarcopenia and Frailty in Hospitalized Older Adults: Recommendations from the Australian and New Zealand Society for Sarcopenia and Frailty Research (ANZSSFR) Expert Working Group

**DOI:** 10.1007/s12603-022-1801-0

**Published:** 2022-05-31

**Authors:** Robin M. Daly, S. Iuliano, J.J. Fyfe, D. Scott, B. Kirk, M.Q. Thompson, E. Dent, K. Fetterplace, O.R.L. Wright, G.S. Lynch, J. Zanker, S. Yu, S. Kurrle, R. Visvanathan, A.B. Maier

**Affiliations:** 1 Institute for Physical Activity and Nutrition, School of Exercise and Nutrition Sciences, Deakin University, Geelong, Australia; 2 Department of Medicine (Endocrinology), University of Melbourne / Austin Health, Heidelberg, VIC, Australia; 3 Australian Institute for Musculoskeletal Science (AIMSS), The University of Melbourne and Western Health, St Albans, Melbourne, VIC, Australia; 4 School of Clinical Sciences at Monash Health, Monash University, Clayton, Victoria, Australia; 5 Department of Medicine-Western Health, Melbourne Medical School, The University of Melbourne, St Albans, Melbourne, VIC, Australia; 6 Adelaide Geriatrics Training & Research. with Aged Care (G-TRAC) Centre, Adelaide Medical School, Faculty of Health and Medical Sciences, University of Adelaide, Adelaide, South Australia, Australia; 7 National Health and Medical Research Council (NHMRC) Centre of Research Excellence: Frailty and Healthy Ageing, University of Adelaide, Adelaide, South Australia, Australia; 8 Basil Hetzel Institute, Woodville South, SA, Australia; 9 Torrens University Australia, Adelaide, South Australia, Australia; 10 Department of Allied health (Clinical Nutrition), Royal Melbourne Hospital, Parkville, VIC, Australia; 11 The University of Melbourne, Department of Critical Care, Melbourne Medical School, Melbourne, VIC, Australia; 12 School of Human Movement and Nutrition Sciences, The University of Queensland, Brisbane, Australia; 13 Centre for Muscle Research, Department of Anatomy and Physiology, School of Biomedical Sciences, Faculty of Medicine, Dentistry and Health Sciences, The University of Melbourne, Melbourne, Victoria, Australia; 14 Aged and Extended Care Services, The Queen Elizabeth Hospital and Basil Hetzel Institute, Woodville South, SA, Australia; 15 Faculty of Medicine and Health, University of Sydney, Sydney, New South Wales, Australia; 16 Department of Human Movement Sciences, @AgeAmsterdam, Amsterdam Movement Science, Vrije Universiteit Amsterdam, Amsterdam, The Netherlands; 17 Department of Medicine and Aged Care, @AgeMelbourne, The Royal Melbourne Hospital, The University of Melbourne, Melbourne, Victoria, Australia; 18 Healthy Longevity Translational Research Program, Yong Loo Lin School of Medicine, National University of Singapore, Singapore, Singapore; 19 Centre for Healthy Longevity, @AgeSingapore, National University Health System, Singapore, Singapore; 20 Institute for Physical Activity and Nutrition, School of Exercise and Nutrition Sciences, Deakin University, 221 Burwood Highway, Burwood, 3125, Melbourne, Victoria, Australia

**Keywords:** Sarcopenia/diagnosis, sarcopenia/therapy, frailty, screening/methods, aged, hospitalization

## Abstract

Sarcopenia and frailty are highly prevalent conditions in older hospitalized patients, which are associated with a myriad of adverse clinical outcomes. This paper, prepared by a multidisciplinary expert working group from the Australian and New Zealand Society for Sarcopenia and Frailty Research (ANZSSFR), provides an up-to-date overview of current evidence and recommendations based on a narrative review of the literature for the screening, diagnosis, and management of sarcopenia and frailty in older patients within the hospital setting. It also includes suggestions on potential pathways to implement change to encourage widespread adoption of these evidence-informed recommendations within hospital settings. The expert working group concluded there was insufficient evidence to support any specific screening tool for sarcopenia and recommends an assessment of probable sarcopenia/sarcopenia using established criteria for all older (≥65 years) hospitalized patients or in younger patients with conditions (e.g., comorbidities) that may increase their risk of sarcopenia. Diagnosis of probable sarcopenia should be based on an assessment of low muscle strength (grip strength or five times sit-to-stand) with sarcopenia diagnosis including low muscle mass quantified from dual energy X-ray absorptiometry, bioelectrical impedance analysis or in the absence of diagnostic devices, calf circumference as a proxy measure. Severe sarcopenia is represented by the addition of impaired physical performance (slow gait speed). All patients with probable sarcopenia or sarcopenia should be investigated for causes (e.g., chronic/acute disease or malnutrition), and treated accordingly. For frailty, it is recommended that all hospitalized patients aged 70 years and older be screened using a validated tool [Clinical Frailty Scale (CFS), Hospital Frailty Risk Score, the FRAIL scale or the Frailty Index]. Patients screened as positive for frailty should undergo further clinical assessment using the Frailty Phenotype, Frailty Index or information collected from a Comprehensive Geriatric Assessment (CGA). All patients identified as frail should receive follow up by a health practitioner(s) for an individualized care plan. To treat older hospitalized patients with probable sarcopenia, sarcopenia, or frailty, it is recommended that a structured and supervised multi-component exercise program incorporating elements of resistance (muscle strengthening), challenging balance, and functional mobility training be prescribed as early as possible combined with nutritional support to optimize energy and protein intake and correct any deficiencies. There is insufficient evidence to recommend pharmacological agents for the treatment of sarcopenia or frailty. Finally, to facilitate integration of these recommendations into hospital settings organization-wide approaches are needed, with the Spread and Sustain framework recommended to facilitate organizational culture change, with the help of ‘champions’ to drive these changes. A multidisciplinary team approach incorporating awareness and education initiatives for healthcare professionals is recommended to ensure that screening, diagnosis and management approaches for sarcopenia and frailty are embedded and sustained within hospital settings. Finally, patients and caregivers' education should be integrated into the care pathway to facilitate adherence to prescribed management approaches for sarcopenia and frailty.

## Introduction

**H**ospitalization rates are highest in older adults, with those aged >65 years accounting for 42% of hospitalizations and 48% of patient days (1, 2). Amongst these hospitalized older adults, the prevalence of sarcopenia and frailty has been reported to be 37% and 47%, respectively (3). Both sarcopenia and frailty are associated with longer hospital stays (4, 5), readmissions (6, 7), institutionalization (8, 9, 10), lower quality of life (QoL) (11) and higher mortality (6). Sarcopenia and frailty often co-occur with malnutrition, which has been reported in 66% of older inpatients (3). The risk of malnutrition is 4.1-fold higher if a patient has sarcopenia and 5.8-fold higher if a patient is frail (3). Moreover, older adults with two or more conditions of either sarcopenia, frailty, or malnutrition are more likely to be hospitalized, and are disproportionally represented in hospitals (3). The high prevalence of sarcopenia and frailty, with or without malnutrition, and their implications for adverse clinical outcomes in hospitalized older adults highlights the need for routine screening and/or assessment and subsequent management of these conditions during hospitalization using best practice, evidence-based approaches. However, there are currently no evidence-informed recommendations based on the available literature related to the screening, assessment and management of both sarcopenia and frailty in older adults within hospital settings. Therefore, the purpose of this paper, prepared by a multidisciplinary expert working group on sarcopenia and frailty from the Australian and New Zealand Society for Sarcopenia and Frailty Research (ANZSSFR), is to provide an up-to-date overview for clinicians and healthcare professionals on the current evidence and provide recommendations for the screening, assessment, and management of sarcopenia and frailty within the hospital setting. It also highlights barriers and potential pathways to implement change to encourage widespread adoption of these evidence-informed recommendations related to sarcopenia and frailty within hospital settings.

## Methods

This manuscript was prepared by a group of 15 clinicians and researchers with expertise in geriatrics, gerontology, dietetics, exercise physiology, occupational therapy, and intensive care. In September 2020, expressions of interest were sought from Australian-based experts within the fields of sarcopenia, frailty and malnutrition to join an ANZSSFR working group to develop recommendations on the screening, diagnosis and management of sarcopenia and frailty for older adults within hospital settings. Those that registered an interest were asked to provide written feedback via email on the proposed aims and structure of the manuscript developed by authors RMD and SI. This was followed by ongoing dialogue (via email) until the aims and structure were finalized, after which three working groups were established. Each working group was asked to provide a narrative review of the available evidence on their topic area, focusing on randomized controlled trials and systematic reviews and meta-analysis where possible, and to draft specific recommendations for further discussion. This was done in an iterative manner over an 8-month period. A complete draft of the manuscript was then sent to all authors for review (multiple rounds of email correspondence over 3–4 months) until 100% agreement (consensus) was achieved for all the recommendations.

## Screening and diagnosis of sarcopenia within the hospital setting

### Definition(s) of sarcopenia, prevalence, and consequences in the hospital setting

Sarcopenia is defined as a progressive and generalized skeletal muscle disease that is characterised by an accelerated loss of muscle mass, strength, and/or function (12, 13, 14). Sarcopenia is a strong predictor of a myriad of adverse outcomes, such as frailty (14), falls and fractures (15) and mortality (16). In hospitalized older adults, sarcopenia is associated with longer hospital stays, higher healthcare costs, greater risk of hospital readmission and mortality (17, 18). In Australia, sarcopenia was formally recognized as a disease in 2019 with an International Classification of Diseases (ICD) code (19). However, since no international consensus has been reached for an operational definition of sarcopenia, the reported prevalence in hospital settings is variable, ranging from 10% to 35% (17, 19, 20, 21, 22), and up to over 50% in post-acute inpatient rehabilitation (23, 24). Sarcopenia often occurs as a comorbid disease in hospitalized older adults (25), which is associated with an even higher risk of institutionalization and mortality (26). Extended periods of bed rest during hospitalization may further contribute to loss of muscle mass and strength (27). In fact, up to 15% of older adults without sarcopenia at hospital admission may meet the criteria for sarcopenia at discharge (28). Furthermore, muscle mass and strength decline may continue even after discharge from acute hospitalization (29), highlighting the need for sarcopenia screening and/or assessment to be included as part of routine care for older hospitalized patients to optimize management.

### Sarcopenia screening tools within the hospital setting

Screening hospitalized older patients for sarcopenia may be useful to help identify those with, or at risk of sarcopenia. One tool that is available and recommended by several key expert groups [European Working Group on Sarcopenia in Older People (EWGSOP) (30), Society on Sarcopenia, Cachexia and Wasting Disorders (SCWD) (31); International Conference on Sarcopenia and Frailty Research (ICFSR) (32)] is the Strength, Assistance in walking, Rise from a chair, Climbing stairs and Falls history (SARC-F) questionnaire, with score of ≥4 predictive of sarcopenia (33). However, a review evaluating SARC-F as a screening tool for sarcopenia from 29 studies (n=21,855 participants) from a range of settings [community-dwelling, geriatric inpatient and outpatient, nursing homes, and long-term care populations], found that SARC-F had low to moderate sensitivity (29–55%) and moderate to high specificity (69–89%), independent of the sarcopenia definition used and population studied (34). Two recent studies involving hospitalized older adults with hip fracture or in geriatric rehabilitation reported mixed findings regarding the sensitivity and specificity of SARC-F for predicting sarcopenia (35, 36). Modified SARC-F versions that include calf circumference (SARC-Calf) (37), thigh circumference (SARC-F+TC), both calf and thigh measures (SARC-F+CC+TC) (38), or age and BMI (SARC-F+EBM) (39), may improve the sensitivity and diagnostic accuracy of the SARC-F, but most available data are based on older adults in the community and not hospitalized older adults. Alternative screening methods are suggested, such as the Mini Sarcopenia Risk Assessment (MSRA) questionnaire, which has two forms, the 5- or 7-items questionnaire, and includes questions related to age, number of hospitalizations in past year, physical activity level, weight loss and regularity of meals (MSRA-5) plus consumption of milk and dairy products and the number of daily meals (MSRA-7) (40). The MSRA-5 has a higher sensitivity (80–90%) compared to SARC-F, but lower specificity (60–80%) based on data from 384 community-dwelling Asian older adults aged 60+ years (40). Collectively, there is currently limited evidence available to inform whether SARC-F, modified versions of this, or other tools, represent a valid screening approach for sarcopenia in hospitalized patients. Thus, screening for sarcopenia in hospitalized older patients is not advisable at this time based on the available data.

### Assessment and diagnosis of sarcopenia in the hospital setting

Most current guidelines recommend the assessment and diagnosis of sarcopenia be based on measurements related to muscle mass, strength, and/or physical function. Although there are multiple operational definitions of sarcopenia, each requires an assessment of at least two or more of these measurements, as summarized in Table 1. In 2019, the ANZSSFR recommended the EWGSOP definition to diagnose sarcopenia in Australia and New Zealand (41). However, this is currently being updated following the introduction of several new definitions of sarcopenia and a recent expert Delphi consensus process by ANZSSFR has recommended that the use of the updated EWGSOP2 definition be used in Australia and New Zealand (42).

**Table 1:** Current operational diagnostic criteria and definitions of sarcopenia

**Definition**	**Muscle strength**	Muscle (lean) mass *	**Physical performance**	**Sarcopenia definitions**
EWGSOP2, 2019 (30)	Handgrip strength Men <27 kg Women <16 kg Five times sit-to-stand >15 seconds	ALM (kg) Men <20 kg Women <15 kg or ALM/Ht2 Men <7.0 kg/m^2^ Women <5.5 kg/m^2^	Gait speed ≤0.8 m/s SPPB ≤8 points 400 m walk ≥6 min	Probable Sarcopenia: low muscle strength Sarcopenia: low muscle strength + low muscle mass Severe Sarcopenia: low muscle strength + low muscle mass and poor physical performance
AWGS, 2020 (43)	Handgrip strength Men <28 kg Women <18 kg	ALM/Ht2 Men <7.0 kg/m^2^ Women <5.4 kg/m^2^	Gait speed <0.8 m/s	Sarcopenia: low muscle mass + low muscle strength OR poor physical performance Severe sarcopenia: low ALM + low muscle strength AND poor physical performance
SDOC, 2020 (44)	Handgrip strength Men <35.5 kg Women <20 kg	Not recommended	Gait speed <0.8 m/s	Sarcopenia: low muscle strength + poor physical performance


EWGSOP2, European Working Group on Sarcopenia in Older People revised definition; AWGS2019, Asian Working Group for Sarcopenia consensus update definition; SDOC, Sarcopenia Definition on Outcome Consortium; ALM, Appendicular lean mass; Ht, height; *Based on dual energy X-ray absorptiometry (DXA); SPPB, short physical performance battery.

Currently, the assessment of sarcopenia is not routinely performed in patients within clinical/hospital settings (45, 46), despite the willingness of older adults and hospitalized patients to counteract sarcopenia (47). Some common barriers to diagnosing sarcopenia in hospital settings (48, 49, 50, 51) are outlined in Table 2 and include the presence of musculoskeletal disorders, acute illness, unmanaged pain or being bed bound. Furthermore, the status of acutely admitted patients often change during hospitalization, which can impact the feasibility, reliability, and ultimately the predictive value of measurements taken upon admission (52).

**Table 2:** Diagnostic tools, clinical challenges and common factors affecting diagnosis of sarcopenia

**Component**	**Tools**	**Clinical challenges that affect the feasibility and/or reliability of measurements**	**Common factors that preclude assessment**
Low muscle strength	Hand dynamometer or chair stand test	Presence of musculoskeletal disorder(s) or acute illness, independent of sarcopenia. Unmanaged pain	Patient Factors • Confusion/dementia • Patient refusal • Pain • Severe acute illness • Aggressive patients • Severe arthritis • Bed bound Staff Factors • Lack of staff awareness of sarcopenia • Not part of clinical assessment Hospital Factors • Lack of funding for investigations/purchasing equipment • Lack of space
Low muscle mass	DXA BIA/BIS CT MRI Ultrasound Anthropometric measurement (calf circumference)	DXA • Not currently performed routinely • Subject to availability BIA/BIS • May be influenced by hydration and co-morbidities, such as cardiac failure or liver failure • Contraindicated in those who have a permanent pacemaker CT/MRI • Costly • Exposure to radiation	
Low physical performance	Usual gait speed Timed-up-and-go (TUG) SPPB (gait speed, balance test and chair stand test) 6-min walk test 400m walk test Stair climb test	Presence of musculoskeletal disorder(s) or acute illness Bed bound Unmanaged pain	


SPPB, Short Physical Performance Battery; DXA, Dual-energy X-ray absorptiometiy; BIA, Bioelectrical Impedance Analysis, Bioelectrical Impedance Spectroscopy; CT, Computed Tomography; MRI, Magnetic Resonance Imaging.

Feasibility studies in acute hospital settings suggest that many patients, except for the critically ill and some neurological/trauma patients, can complete muscle strength and physical performance measurements (48, 49, 50, 51). However, assessment of muscle mass has the lowest completion rate (50), likely due to a lack of appropriate equipment available to measure muscle (lean) mass or its surrogates (46). EWGSOP2 recommends that those with confirmed low muscle strength be classified as probable sarcopenia (30), and the Sarcopenia Definition on Outcome Consortium (SDOC) (44) recommends only assessment of muscle strength and physical performance for sarcopenia diagnosis. In the absence of a measure of muscle mass or its surrogates, a diagnosis of probable sarcopenia can be made based on muscle strength alone (30). To diagnose sarcopenia in settings where no muscle mass diagnostic methods are available, EWGSOP2 (30) and consensus recommendations from Singapore (53) recommend that calf circumference may be used as a diagnostic proxy. Although a specific diagnostic calf circumference cut-point(s) to define low muscle mass was not defined by the EWGSOP2, they reported that a cut-off of <31 cm can predict performance and survival in older adults (30). However, when calf circumference measures were validated against DXA measures of appendicular lean mass (54, 55, 56) and assessed in a large cohort (n=17,789) of healthy adults aged ≥18 years (56), cut-offs of ≤33 cm for women and ≤34 cm for men were recommended to define low muscle mass. In older hospitalized patients, low muscle mass identified by calf circumference was associated with hospital readmissions (57), nutritional risk (58, 59) and mortality (60). However, caution is required when measuring calf circumference in patients with oedema or obesity as it may led to false-negative results (53).

## Key recommendations for screening and assessment of sarcopenia in hospital settings

1. There is currently insufficient evidence to support the use of any specific screening tool for sarcopenia within the hospital setting, and thus the assessment of sarcopenia (without screening) is recommended.
2. Hospitalized patients aged 65 years and older, or those with conditions or circumstances (e.g., comorbidities) that may increase the risk of sarcopenia at a younger age, should be assessed for probable sarcopenia and/or sarcopenia during hospital admission.
3. Probable sarcopenia and sarcopenia should be diagnosed based on an assessment of muscle strength and appendicular lean (muscle) mass. An expert Delphi consensus process by ANZSSFR currently recommends using the EWGSOP2 revised definition (42). These guidelines firstly recommend an assessment of muscle strength, which if low indicates probable sarcopenia, followed by an assessment of muscle quantity (mass) quantified by DXA or BIA, which if low confirms sarcopenia. An assessment of physical function, which if impaired, can be used to indicate severe sarcopenia.
4. In the absence of availability of muscle mass quantification techniques such as DXA and BIA, calf circumference may be used as a surrogate estimate of muscle mass in patients without oedema or obesity, with cut-offs of ≤33 cm for women and ≤34 cm for men to be considered to define low muscle mass.
5. Patients who meet the criteria for probable sarcopenia, sarcopenia or severe sarcopenia should be investigated for causes of low muscle strength, mass and/or function (e.g., chronic/acute disease or malnutrition), and treated accordingly.

## Screening and diagnosis of frailty within hospital settings

### Definition(s) of frailty, prevalence and consequences in the hospital setting

Frailty is a complex geriatric condition often defined as a diminished physiological reserve across several organ systems that results in increased vulnerability to stressors (61, 62). Frailty in hospitalized older adults is associated with falls, delirium, prolonged and recurrent hospitalization, decreased quality of life (QoL), malnutrition, functional decline, admission to residential aged care, and mortality (62, 63, 64). Various tools exist to either screen or diagnose frailty in the hospital setting, but their validity and feasibility depend on several factors, such as the population of interest (e.g., elective or acute admission) and the timing of assessment (e.g., immediately upon admission or at discharge) (65, 66).

### Frailty identification within the hospital setting

The frailty status of older patients at admission is predictive of a range of adverse outcomes including inpatient mortality, length of stay, and discharge to residential aged care (7, 67). Thus, the identification of frailty in hospitalized older adults is recommended to guide clinical judgement and to prioritize care (61). There are several validated frailty screening tools available; however the Clinical Frailty Scale (CFS) (68), the Frailty Index (70) and the Hospital Frailty Risk Score (HFRS) are the most commonly used in acute settings (71) (Table 3), whilst the simpler to use FRAIL scale (69) is applied in the community setting and perhaps useful prior to discharge. The CFS, a clinical judgement-based tool that evaluates specific domains (e.g., comorbidity, function, and cognition) to generate a frailty score ranging from 1 (very fit) to 9 (terminally ill), has been identified as one of the most feasible frailty screening tools for use in acute settings (68, 72). More recently, a classification tree has been proposed to enable more reliable classification of the CFS and enable the wide translation of the CFS into clinical practice (73). The FRAIL scale is a short frailty screening instrument based on patient self-reporting and has a good predictive validity for mortality but studies in hospital settings are rare (69, 74). The HFRS, which is demonstrated to relate to increased mortality risk (75), is estimated by deriving a score among 109 diagnostic codes of the International Statistical Classification of Diseases and Related Health Problems, 10th Revision (ICD-10) that have been assigned a score based on how well each code predicts frailty (71). Finally, the Frailty Index which assesses frailty in relation to the accumulation of health deficits, is predominantly an assessment tool derived from a comprehensive geriatric assessment (CGA), however it may be used for screening as some electronic medical records automatically generate a Frailty Index score as demonstrated in primary care in England (70, 76) Therefore, based on the available evidence routine screening for frailty is recommended for all adults aged 70 years and over within the hospital setting utilizing one of the above validated tools. Importantly, all health practitioners undertaking frailty screening should first receive appropriate training (61).

**Table 3:** Common frailty screening and assessment tools that can be used in hospital settings.

**Tool**	**Classification or scoring**
Clinical Frailty Scale (CFS) (68)	Based on clinical judgement of an individual's dependency level and health state according to a nine-point clinical scale with associated pictures ranging from very fit (Category 1) to terminally ill (Category 9) 1. Very Fit — People who are robust, active, energetic, and motivated. These people commonly exercise regularly. They are among the fittest for their age. 2. Well — People who have no active disease symptoms but are less fit than category 1. Often, they exercise or are very active occasionally e.g., seasonally 3. Managing Well — People whose medical problems are well controlled but are not regularly active beyond routine walking. 4. Vulnerable — While not dependent on others for daily help, often symptoms limit activities. A common complaint is being ‘slowed up’, and/or being tired during the day. 5. Mildly Frail — These people often have more evident slowing and need help in high order instrumental activities of daily living (finances, transportation, heavy housework, and medications). Typically, mild frailty progressively impairs shopping and walking outside alone, meal preparation, and housework. 6. Moderately Frail — People need help with all outside activities and with keeping house. Inside, they often have problem with stairs and need help with bathing and might need minimal assistance (cuing, standby) with dressing. 7. Severely Frail — Completely dependent for personal care, from whatever cause (physical or cognitive). Even so, they seem stable and not at high risk of dying (within ∼ 6 months). 8. Very Severely frail: Completely dependent, approaching end of life. Typically, they could not recover from even a minor illness. 9. Terminally Ill — Approaching the end of life. This category applies to people with a life expectancy <6 months who are not otherwise evidently frail.
FRAIL scale (69)	Includes five components: Fatigue — How much of the time during the past 4 weeks did you feel tired A — All or most of the time = 1; B — Some, a little or none of the time = 0 Resistance — In the last 4 weeks by yourself and not using aids, do you have any difficult walking up 10 steps without resting? Yes = 1; No = 0 Ambulation — In the past 4 weeks by yourself and not using aids, do you have any difficulty walking 300 metres or one block? Yes = 1; No = 0 Illness — Did you doctor ever tell you that you have: hypertension, diabetes, cancer (not a minor skin cancer), chronic lung disease, heart attack, congestive heart failure, angina, asthma, arthritis, kidney disease? 0–4 answers = 0; 5–11 answers = 1 Loss of weight — Have you lost more than 5 kg or % of your body weight in the past year? Yes = 1; No = 0 Scoring: Robust = 0; Pre-frail = 1–2; Frail>3
Frailty Index (70)	Calculated by counting the number of deficits from a total list of potential deficits for that person. For example, if an individual has 10 deficits from a total of 40, the index is 0.25.
Hospital Frailty Risk Score (71)	This is calculated using 109 diagnostic codes from the International Statistical Classification of Diseases and Related Health Problems, 10th Revision (ICD-10), where each diagnostic code is assigned a score based on frailty prediction. Scoring: Low risk <5; Intermediate risk (5-15) and high risk >15.
Frailty Phenotype (77)	Measures deficits in five domains • Weight loss — self-reported unintentional weight loss or decreased appetite • Exhaustion — self-reported energy levels • Physical activity — frequency of moderate intensity activity • Muscle strength — measured grip strength with dynamometer • Walking speed — self-reported slow speed or measured slow gait Frail if three or more of the above are present.

### Assessment and diagnosis of frailty in the hospital setting

Frailty assessments include the Frailty Phenotype or the abovementioned Frailty Index (accumulation of deficits) (70, 77, 78). The Frailty Phenotype defines frailty as the presence of three or more of the following: weakness, slow gait speed, low physical activity, exhaustion, and unintentional weight loss (77, 79). Pre-frailty is defined when only one or two of these physical characteristics are present. The Frailty Index accounts for the cumulative deficits present in an individual across a range of physical and psychological variables (70, 78), with deficits of >21% (80) or >25% (81) (of at least 30) commonly used to represent frailty. While the prevalence of frailty is influenced by the assessment tool used (ranging from 9% to 48%), there is evidence that the Frailty Index (cumulative deficit model) typically classifies more individuals as frail compared with the Frailty Phenotype approach (82). Nevertheless, older people admitted to hospital are more likely to be frail, with frailty phenotype estimated between 40% and 66% (80, 83, 84), highlighting the need for frailty screening and assessment to become part of routine clinical practice.

Comprehensive geriatric assessment (CGA) and management is a critical process in addressing frailty in hospitalized older adults (32, 85). CGA is a personalized process covering a range of health and functional domains and, in hospital, is typically carried out by a multidisciplinary team who works collaboratively in the development and implementation of a treatment plan (85). The involvement of patients and carers in setting goals is an important part of this approach. Despite a definitive scope and content of a CGA, the World Health Organisation's (WHO) Integrated Care for Older People (ICOPE) offers a useful framework for approaching the screening, assessment, and management of older people, with the aim of reversing or slowing losses in intrinsic capacity, defined as the composite of all the physical and mental capacities of the person (86). This approach recognizes that conditions are often interrelated and require an integrated approach to management. Regular review of frailty and associated conditions is important as the recency of assessment is prognostically most useful, reflecting the dynamic nature of frailty (87). Some of the key domains and associated conditions that should be reflected in a biopsychosocial CGA include cognitive decline, limited mobility, sarcopenia, malnutrition, visual impairment, hearing loss, depressive symptoms, social care and support, caregiver support, delirium, polypharmacy, and chronic conditions (88).

The CGA is aimed at identifying a range of reversible factors that can be prioritized and addressed to optimize care during admission. For severely frail individuals, this may include consideration of palliative options. While the full implementation of a personalized care plan may not be achievable in the acute setting, a detailed assessment and management plan, developed by a multidisciplinary hospital team, can be carried over to the primary care setting for longer term follow-up as well as through referrals for aged care support. The key to successful reablement is compliance with ongoing therapy be it physical, nutritional, or a combination of treatments. The available evidence indicates that good compliance is achieved when ongoing support and monitoring is provided so ideally contact with the patient is continued after discharge until the desired goal it reached (89, 90).

## Key recommendations for screening and assessment of frailty in hospital settings

• All hospitalized patients aged 70 years and older, or those with conditions (e.g., comorbidities) that may increase the risk of frailty at a younger age, should be screened for frailty using the Clinical Frailty Scale (CFS), the FRAIL scale, the Hospital Frailty Risk Score or the Frailty Index, depending on the resources available and objectives for each specific clinical setting.
• Patients screened as positive for frailty (or pre-frailty) should undergo further clinical assessment for frailty using the Frailty Phenotype, Frailty Index or by using information collected from a Comprehensive Geriatric Assessment (CGA).
• Patients identified as frail should receive follow-up by a health practitioner(s) for a multi-disciplinary CGA and development of an individualized care plan that is reviewed and revised as required.

The following sections will provide an overview of the latest evidence related to the role of nutrition, exercise and multifaceted and pharmacological interventions for the management of sarcopenia and frailty in the hospital setting.

## Nutritional management strategies for sarcopenia and frailty in the hospital setting

The aim of nutritional management for sarcopenia and frailty in hospitalized older adults is to stabilize their condition during the acute phase and optimize nutritional status through the recovery phase. The primary focus is to prevent loss of muscle mass and maintain physical function and health-related QoL (91). Best practice guidelines recommend routine screening for malnutrition and implementing supportive measures, such as providing a pleasant eating environment, assistance at mealtimes, and providing energy-dense and high-quality protein rich foods (91, 92, 93). However, when such measures are insufficient in the hospital setting, and where a patient's nutritional needs are not met, food modification, dietetic counselling, oral or enteral nutrition feeding/supplementation (especially if nutritional support is <75% of requirements over one week), or parenteral nutrition (in the case of gastrointestinal dysfunction) all need to be considered (91). For malnourished patients, nutritional interventions that include a food-first approach with oral nutrition support, and/or enteral nutrition were found to be associated with increased energy and protein intakes, reduced mortality, fewer hospital readmissions and greater weight gain (94, 95). With regards to sarcopenia and frailty, the following section will provide an overview of the current evidence for the role of nutrition for the management of these conditions within hospital settings.

The cornerstone to nutritional interventions for older hospitalized patients with or at risk of sarcopenia or frailty and/or with malnutrition is the provision of adequate energy and protein (92, 96). The European Society of Parenteral and Enteral Nutrition (ESPEN) guidelines for clinical nutrition in geriatrics recommend the provision of daily oral nutrition supplements containing 400 kcal and 30 g of protein to older hospitalized patients with or at risk of malnutrition and with chronic conditions (92). Indirect calorimetry is the gold standard to determine energy expenditure; however, it is not routinely available nor practical in hospital settings (97, 98). In that case, validated equations, such as the Schofield equation, with appropriate stress and activity factors (99), or weight-based equations can be used (97). For hospitalized geriatric patients, the ESPEN guidelines recommend an energy intake of at least 30 kcal/kg body weight, however this may be as high as 38 kcal/kg body weight in underweight older patients (92). These values are intended as a guide only and should be individualized based on regular monitoring of the patient's weight, fluid status, and acceptance and tolerance of nutritional support. At times of critical illness, energy requirements should not exceed this value, as this may cause additional catabolic stress (98, 100). In addition, it is recommended that in the first 3–5 days upon admission, energy provision does not exceed 70% of measured energy expenditure or 20–25 kcal/kg body weight (98).

Adequate protein intake is essential to promote muscle protein synthesis (MPS), which is central to prevent or minimize loss of muscle that typically occurs during hospitalization and/or with disuse (101, 102). For older hospitalized patients, current guidelines recommend a protein intake of 1.2–1.5 g/kg body weight per day (91, 93, 103), or up to 2 g/kg body weight per day for those with critical illness or clinical conditions (e.g., burns, multi-trauma, and obesity) (98, 103, 104). Daily protein provision should be calculated using the patient's actual body weight or adjusted body weight for obese individuals [ideal body weight + 25% excess weight (actual body weight − ideal body weight] (98, 105). Despite these recommendations, evidence to support the benefits of protein alone or as part of a ONS to prevent or attenuate muscle loss in older hospitalized patients with or at risk of sarcopenia or frailty is limited. The most comprehensive summary of the evidence to date was reported in a 2019 systematic review of randomized controlled trials (RCTs) which identified six nutrition interventions targeting markers of sarcopenia in older (>65 years) adults in hospital, three of which included an enhanced exercise program (96). The nutrition interventions ranged from two weeks to 12 months and provided an additional 10–40 g/d of protein plus varying doses of energy and other macro- and micro-nutrients (96). Meta-analysis of five studies showed that the nutritional interventions had a positive effect on grip strength (mean difference 1.97 kg) compared to controls (96). There was insufficient data for meta-analysis on muscle mass or function, but two of the four studies that measured lean mass reported a preservation compared to controls (96). In critically ill patients there is some evidence that protein supplementation to current recommendations may attenuate loss of muscle mass compared to standard care (102, 106), but further research is needed to determine if provision of dietary protein alone or as part of an ONS (and at what dose and frequency) may be effective to attenuate (or prevent) loss of muscle in hospitalized older adults with sarcopenia or frailty. In non-hospitalized older adults, it has been recommended that daily protein intake should be divided evenly across the three main meals at a dose of 0.4 g/kg/meal to promote MPS throughout the day (53, 103). However, the limited short-term trials examining the effects of the frequency of protein consumption and per-meal dose on muscle-based outcomes in older hospitalized patient have reported mixed findings (107, 108).

The role of specific nutrients including essential amino acids (EAAs), particularly the branched chain amino acid leucine which acts as the ‘trigger’ for MPS, beta-hydroxy beta-methylbutyrate (HMB), a metabolite of leucine that can promote MPS and inhibit muscle protein breakdown, vitamin D, creatine and omega-3 fatty acids for the management of sarcopenia and frailty has not been well studied in hospitalized older patients, with the limited evidence inconclusive (109, 110, 111, 112, 113). However, there is evidence from several RCTs the multi-nutrient oral supplemental nutrition which includes high quality protein, HMB, vitamin D and/or other macro- and micro-nutrients may play a role in reducing the risk of sarcopenia and other related hospital complications in older hospitalized patients with or at risk of sarcopenia or frailty, recovering from hip fracture and/or with malnutrition (114, 115, 116, 117). For instance, the NOURISH (Nutrition effect On Unplanned Readmissions and Survival in Hospitalized patients) trial involving 652 malnourished older hospitalized adults (mean age 78 years), randomized to either multi-nutrient supplementation (twice daily, 350kcal, 20g protein, 160IU vitamin D and 1.5g HMB) or placebo during hospital stay and following discharge, observed a reduction in 90-day mortality (RR 0.49, 95%CI 0.27–0.90) and improved nutritional status (OR 2.04, 95%CI 1.28–3.25), but no difference in hospital readmission (114). Further analysis showed that this multi-nutrient supplement also had a positive effect on handgrip strength (115). Several other RCTs in older patients (including those who were malnourished and sarcopenic) recovering from hip fracture also found that oral nutritional supplementation with protein, HMB and vitamin D was associated with greater muscle strength, a shorter immobilization period, accelerated wound healing and a maintenance of appendicular lean mass (114, 115, 116, 117). The EFFORT (Effect of early nutritional support on Frailty, Functional Outcomes, and Recovery of malnourished medical inpatients) clinical trial in over 2000 patients (mean age 72 years) at risk of malnutrition demonstrated that individualized nutrition therapy to achieve energy, protein and micronutrient requirements compared to standard hospital food significantly reduced adverse clinical outcomes (defined as a composite of all-cause mortality, admission to intensive care, non-elective hospital readmission, major complications, and decline in functional status at 30 days) as well as mortality, functional decline at day 30 and activities of daily living (ADL) (118). In this study the intervention group achieved a modest daily increase of 290 kcal in energy and 10 g protein compared to controls. While further research is required to determine the effectiveness of high protein, multi-nutrient oral nutritional supplements on sarcopenia and frailty related outcomes in older hospitalized patients, it is important that all older patients are screened or assessed for malnutrition and micronutrient deficiencies (e.g., vitamin D deficiency) and treated appropriately. When oral nutrition support is inadequate (<75% of requirements over one week) or not feasible, enteral nutrition support and/or parenteral nutrition should be considered (92, 98, 119).

## Exercise and mobility interventions for managing sarcopenia and frailty in hospitalized older adults

High-level evidence from RCTs (120, 121, 122, 123) and meta-analyses (124, 125) indicate that in-hospital exercise interventions for older adults, including patients with sarcopenia and/or frailty, involving progressive resistance training (PRT) or multicomponent programs incorporating PRT with balance/gait training, are safe, feasible, and effective for preventing functional decline during hospitalization (122, 124, 126). For instance, a meta-analysis of seven RCTs examining the effects of resistance exercise interventions [typically 20–40 minutes per session, 5–7 days per week and often twice daily (morning and evening)] in 2498 acute hospitalized older adults reported significant increases in muscle strength (mean difference: grip strength 2.5 kg; leg press one-repetition maximum 19.3 kg), muscle power (mean difference: leg press, 29.5 watts) and function (mean difference: timed-up-and-go 3.4 seconds; SPPB 1.29 points) at discharge compared to usual hospital care (125). There is some evidence that combining PRT with targeted balance training for 12 weeks led to greater improvements in ADL, gait speed, grip strength and SPPB scores compared to PRT alone (121). A systematic review of 10 RCTs among older adults aged >75 years with prefrailty or frailty also reported that exercise interventions combining resistance and balance exercises improved physical symptoms of frailty (including poor mobility, balance, strength, and/or muscle mass) in primary and secondary care units (127). Collectively, these findings indicate that hospital-based resistance-based and multicomponent exercise programs are effective for improving muscle strength and physical function in hospitalized older patients.

Various hospital-based initiatives have aimed to improve in-hospital patient mobility levels by getting patients out of bed, standing, and ambulating to limit disuse-related functional decline during hospitalization. These include the End PJ Paralysis (128) and the MOVE ON (Outcomes of Mobilisation of Vulnerable Elders in Ontario) interventions that focus on early mobility assessment (within 24 hours of admission) and progressive mobilization (129), but the effectiveness of these and similar interventions (130) has been inconclusive. Other interventions using information booklets together with physiotherapy-led advice (131) or programs involving regular walking combined with rising from a chair (1–3 times per day, ∼20 minutes per session) (123) have reported positive effects by limiting functional decline or disability associated with hospitalization. However, systematic reviews examining interventions aimed at alleviating decline in physical performance (132) or muscle loss (133) in hospitalized older adults reported limited effectiveness of in-hospital mobility programs alone so are unlikely sufficient for preventing and/or treating sarcopenia. To date, there is no intervention of this type that is specifically targeted at older patients living with frailty. Nonetheless, early and regular mobilization during hospitalization (134) should be encouraged for older patients due to its potential benefits for limiting functional decline. However, there is insufficient evidence currently to support mobilization initiatives as a standalone intervention to counter loss in muscle mass and strength during hospitalization.

## Multifaceted interventions combining exercise and nutrition in hospitalized older people

Clinical guidelines by the International Clinical Practice Guidelines for Sarcopenia (ICFSR) for the management of sarcopenia (32) and frailty (61) recommend high protein nutritional interventions in combination with exercise training (resistance with or without other exercise modalities). Despite some inconsistencies in the findings from studies evaluating the effectiveness of multifaceted exercise and nutrition approaches (32, 96, 111, 135, 136, 137, 138), an 8-week, double-blinded, RCT in 140 older (≥ 65 years) hospitalized sarcopenic adults demonstrated that the consumption of a multi-nutrient drink (twice daily, 20 g whey protein, 2.8 g leucine, 800 IU vitamin D, vitamins, minerals, and fibres) enhanced the effects of a supervised multicomponent exercise (rehabilitation) program (5 days per week, 20–30 minutes, resistance, gait and balance training) on gait speed, whole-body and appendicular muscle mass, grip strength, physical function (SPPB, timed-up-and-go, chair stand), ADLs, and cognitive function (139). Several systematic reviews and meta-analyses of interventions in prefrail/frail, malnourished, and/or sarcopenic older individuals hospitalized with acute and chronic conditions (96, 111, 135, 136, 137, 140) have also found some evidence for a positive effect of protein/essential amino acid or other oral nutritional supplementation combined with exercise/rehabilitation for improving muscle mass, strength and function, and reducing frailty and frailty-related indicators. Based on the available evidence, it is recommended that multifaceted exercise and nutrition approaches be adopted for the management of sarcopenia and frailty in hospitalized older patients.

## Pharmacological interventions for the management of sarcopenia and frailty

Few pharmacotherapies have been developed specifically for sarcopenia, with most being used to attenuate muscle wasting and weakness associated with conditions such as muscle trauma, metabolic and neuromuscular disease(s) and cancer. Available reviews include results from trials on anabolic approaches to increase muscle (lean) mass and improve muscle strength, such as testosterone replacement, targeting myostatin with neutralizing antibodies, targeting the activin receptor with an antagonist, or treating with selective androgen receptor modulators (141). Despite preclinical studies showing potential of these approaches for attenuating muscle loss or enhancing lean mass, translation to clinical trials is modest and fails to meet clinically relevant outcomes related to muscle strength and physical performance or frailty (142, 143). In addition, for many pharmacotherapies for sarcopenia, especially testosterone or growth hormone replacement to promote skeletal muscle anabolism, the relative risk-to-benefit ratio of these approaches must be considered and may preclude widespread application (144, 145). For example, although testosterone treatment may improve muscle strength in community dwelling older adults (146), long-term effects on disease susceptibility such as prostate cancer and cardiovascular events must be considered. These effects may be offset through consideration of co-treatments to address off-target effects or modifying the duration of treatments.

Other pharmacotherapies for sarcopenia include non-steroidal anti-inflammatory drugs (NSAIDs) to target inflammatory cytokine signalling, drugs such as mTOR inhibitors, and BIO101 or metformin, to address age-related metabolic dysfunction (142). Approaches targeting components of antiapoptotic pathways using senolytic drugs or ‘senotherapeutics’ that kill senescent cells or inhibit the senescence-associated secretory phenotype, have received considerable attention over the last decade for their potential application to treat age-related diseases (147). Despite promising therapeutic benefits, side effects of senolytics have been identified in some studies, including potential mitochondrial impairments (148) and potential cardiotoxicity (149). Furthermore, the evidence regarding the role of cellular senescence in human muscle and disease processes is limited compared to other organ systems (150).

At present, there are no safe and effective drugs recommended (or available) as frontline pharmacological therapy for sarcopenia and frailty (143). While we must await the outcome of future clinical trials, a consensus of the conduct of clinical trials for sarcopenia has been formulated (151).

## Key recommendations related to nutrition, exercise, multifaceted and pharmacological interventions for hospitalized older patients with or at risk of sarcopenia or frailty

• Older hospitalized patient identified as having probable sarcopenia, sarcopenia or frailty, with or without malnutrition, should be assessed and monitored by a dietitian to determine the most appropriate nutritional support and correct any deficiencies. Nutrition support interventions should be escalated in patients who do not meet nutritional goals during the first 3–5 days of admission.
• Nutritional interventions delivered via whole foods that incorporate additional energy and/or protein or high protein, multi-nutrient ONS should aim to provide at least 30 kcal/kg energy and 1.2–1.5 g/kg protein per day to hospitalized patients with malnutrition, sarcopenia or frailty; however, these should be adjusted according to the patient (e.g., obesity, critical illness) and the clinical setting (e.g., ICU).
• Multicomponent exercise programs prescribed and supervised by qualified healthcare professionals incorporating elements of resistance, challenging balance, and functional training mimicking ADLs should be implemented as early as possible following hospital admission to limit functional decline and for management of sarcopenia or frailty.
• To optimize muscle health and function a multicomponent exercise program combined with high protein, multi-nutrient nutritional support to ensure sufficient energy, protein, and other macro/micro-nutrient is recommended.
• There is insufficient evidence to recommend any pharmacological agents for the treatment of sarcopenia or frailty.

## Pathway to change for the management of sarcopenia and frailty for healthcare professionals working within hospital settings

Translation of evidence into person-centred hospital care is a ubiquitous challenge in healthcare globally (152). Despite significant advances in knowledge in the fields of sarcopenia and frailty over the past two decades, organisational, political and cultural resistance to change can create a challenging environment for clinicians, allied healthcare professionals and researchers aiming to translate evidence into best practice in hospitals. The following section highlights some of the key barriers to change and offers potential solutions when implementing a sustainable best practice for sarcopenia and frailty management in hospitals.

### Barriers to change practice within hospital settings

A range of institutional, professional, and patient/caregiver factors may be potential barriers to sarcopenia and frailty screening, diagnosis and management within hospitals (43). These include hospital guidelines and practices which may result in conflicting priorities, inadequate resourcing, and challenges in coordinating a multidisciplinary team response (43). A lack of professional awareness, education/training and knowledge about sarcopenia and frailty screening, diagnosis and its management (46, 153), and a narrow focus on the acute presenting condition, can also make change more challenging (43). In addition, issues related to availability of equipment, time constraints and lack of collaboration have also been identified as barriers that can hinder the diagnosis and management of sarcopenia (45). Finally, poor knowledge and adherence from patients and caregivers due to a lack of understanding about sarcopenia and frailty and how these conditions can be managed, and their involvement in care planning can pose a barrier to change and implementation of evidence-based practice (43).

Structural characteristics can also serve as barriers to change, and include i) dysfunctional characteristics found in most organizations, such as societal norms and values that provide stability and stasis; ii) dysfunction specific to the healthcare sector, such as the roles and responsibilities of healthcare professionals in actioning change while simultaneously being the core employees in the hospital, and iii) dysfunctional dimensions of politically managed organizations, such as non-optimizing and non-rational actions resulting from decision-making in all sectors of public policy (154). Contextual sources of resistance to change, or internal characteristics of hospitals, also need to be considered by hospital leaders in the context of structural characteristics. Firstly, it is important to acknowledge that organizing and providing high-quality care is complex. Within a hospital system, coordinating best care for patients with sarcopenia or frailty requires a synchrony of the multidisciplinary clinical team, managers, logistics staff, and service delivery staff (particularly food services). Secondly, privatisation within hospitals, such as privately contracting the delivery of public services (e.g., food services, cleaning, pathology), have not been proven to lead to improved outcomes (155). Finally, staff anxiety (both managerial and clinical) may be generalized or specific due to competing priorities when caring for patients. Anxiety may arise due to a lack of clinical knowledge or clear guidelines coupled with low levels of perceived importance (154). This is understandable in the context of an ever-changing evidence base alongside a lack of consensus regarding definitions, screening and assessment methods and management recommendations for sarcopenia and frailty.

### Potential solutions for implementing change within hospital settings

Each healthcare setting and network has a unique culture with variable willingness and ability to adapt to change. However, three main components for overcoming barriers to change and successful implementation of sarcopenia and frailty diagnostics and interventions can be considered: i) initial success; ii) sustaining, and iii) spreading the change. The initial success of practice change requires an engagement from all stakeholders and knowledge of the barriers to change within the organizational context (156). Stakeholders include all members of the multidisciplinary team, operational staff, logistics, and patients, who through education and involvement in the process, may assist in supporting the rationale for change. Sufficient time should be dedicated to developing and testing practice strategies in partnership with key personnel before imbedding change into practise. Overcoming these barriers will require healthcare professional and patient/caregiver education/training and awareness initiatives on the importance of sarcopenia and frailty as a key component in acute care. There is also a need for system modification so that patients are screened and/or diagnosed for sarcopenia and frailty and educated on how best to manage their condition. Furthermore, identification of responsibility for screening, assessment and management that includes a multidisciplinary team approach involving clinicians, nurses, allied health professionals (dietitians, exercise physiologists, physiotherapists) and other relevant healthcare professionals is needed (43, 53). It is of upmost importance that frailty and sarcopenia assessment and interventions are integrated in key components of care models, such as the CGA (32, 85). Frailty and sarcopenia assessment and interventions should also be an integrated part of care models such as the ‘Hospital Elder Life Program’ (HELP) (157), ‘Nurses Improving Care for Health System Elders’ (NICHE) (158) and the ‘Acute Care for Elders (ACE) unit’, which was the first Senior Friendly Hospital (SFH) program developed in the USA in 1990 (159). Older patients treated in ACE units have improved physical function, and shorter lengths of stay and costs compared to usual care (160). Feasibility is also a critical consideration in addition to validity and reliability when selecting a tool(s) for the screening and/or diagnosis of sarcopenia and frailty. Once success is observed, plans for ongoing monitoring should be implemented with continual strategic review to ensure flexible adaptation to organizational needs (156).

**Figure 1:**
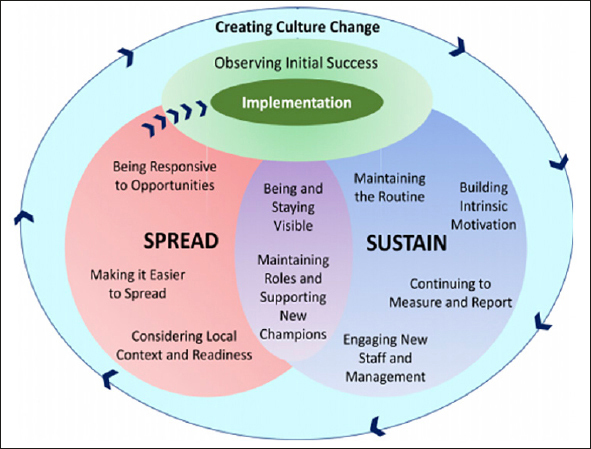
The Sustain and Spread Framework: Once there is initial implementation success, strategies are used to sustain and spread the successful change Taken from Laur C et al. (156)

Sustainability of practice improvements is core to enhancing patient care long-term (156). When sustainable practices are spread, organization-wide culture change can flourish (156). The Sustain and Spread model, examined in Canada, has shown effectiveness in fostering culture change in nutrition care leading to positive impacts on patients (156). This model may also apply to the implementation of sarcopenia and frailty best practice. Figure 1 illustrates the Spread and Sustain model designed to lead to organisational culture change.

Change Champions — interested staff members whose role is to educate, motivate, and implement change, (161) are key for a sustainable change. An example may be a Champion nurse who provides formal/informal education to team members about the need for applying the frailty screening tool to inpatients, using improved patient care and outcomes as the motivator. While barriers to change in hospital settings are complex and extensive, by adopting a strategic approach that capitalizes on existing human resources, recognizes barriers, and employs a sustain and spread strategy, culture change aiming to improve patient care is possible.

## Key recommendations related to implementing change for the management of sarcopenia and frailty for hospitals and healthcare professional working within a hospital setting

• Organization-wide approaches within hospitals are needed to support strategies to identify, prevent, or manage sarcopenia and frailty. The Sustain and Spread model can be used to steer organisational culture change, with the help of champions within the hospital setting to strive for the routine screening, assessment and management of sarcopenia and frailty.
• A multidisciplinary team approach incorporating clinicians, nurses, allied health professionals (dietitians, exercise physiologists, physiotherapists, occupational therapists) and other relevant healthcare professionals is recommended to ensure that screening, diagnosis and management approaches for sarcopenia and frailty are embedded and sustained within hospital settings.
• Awareness and education initiatives are needed to upskill relevant healthcare professionals working with older hospitalized patient on screening, diagnosis and management approaches for sarcopenia and frailty.
• Patients and caregivers' education should be integrated into the care pathway to facilitate uptake and adherence to prescribed management approaches for sarcopenia and frailty.

## Concluding Remarks

Sarcopenia and frailty are highly prevalent in older hospitalized patients, which are associated with a myriad of adverse clinical outcomes, highlighting the need for routine screening and/or assessment and subsequent management using best practice, evidence-based approaches. Our evidence-informed recommendations are intended to serve as a platform to provide guidance and facilitate change within hospital settings with regards to the uptake, dissemination and implementing of best practice screening, assessment and management approaches for sarcopenia and frailty in older hospitalized patients. This paper also intends to stimulate further research in this area to address current gaps in knowledge with the aim to provide the necessary evidence to ensure that sarcopenia and frailty screening, diagnosis and management become embedded into routine clinical practice in all hospitals. It is acknowledged however, that the multidisciplinary panel of experts from the ANZSSFR expert working group on sarcopenia and frailty developed their consensus recommendations based on a narrative review of the latest evidence from RCTs, systematic reviews and meta-analyses and current international guidelines.
